# Antiproliferative Properties of *Papaver rhoeas* Ovule Extracts and Derived Fractions Tested on HL60 Leukemia Human Cells

**DOI:** 10.3390/molecules25081850

**Published:** 2020-04-17

**Authors:** Elisa Ovidi, Valentina Laghezza Masci, Stefania Garzoli, Gabriella Gambellini, Saran Vittoria Keita, Daniele Zago, Giovanni Turchetti, Lorenzo Modesti, Antonio Tiezzi

**Affiliations:** 1 Department for the Innovation in Biological, Agro-food and Forestal systems, Tuscia University, 01100 Viterbo, Italy; 2 Department of Drug Chemistry and Technology, Sapienza University, 00100 Rome, Italy; 3 Center of Electron Microscopy, Tuscia University, 01100 Viterbo, Italy

**Keywords:** *Papaver rhoeas*, ovules, plant compounds, cytotoxic activity, GC-MS

## Abstract

*Papaver rhoeas* plant is common in many regions worldwide and contributes to the landscape with its red flower. In the present study we first carried out morphological investigation by optical and scanning electron microscopy of the ovules within the ovary. After ovules’ isolation we prepared extracts to test possible cytotoxic activities on HL60 leukemia human cells and investigated the extracts using thin-layer chromatography (TLC) and gas-chromatography/mass spectrometry (GC-MS). *P. rhoeas* ovules showed an elongated, round shape and the presence of ordered sculptures on the ovule surface. The ovule extracts showed cytotoxic activity on HL60 human cells mainly found in some TLC-isolated spots. Compounds consisting of active spots were identified by GC-MS investigations. Our findings on the *P. rhoeas* ovule compounds open perspectives for further investigations of TLC-isolated spots on other human cancer cell lines.

## 1. Introduction

Since ancient times, plants and their components have been used by humans as resources to improve the quality of life. Their use as pharmaceuticals shifted from empirical applications to detailed scientific uses [1] and recently the search for plant compounds for pharmaceutical development has significantly increased [2].

In our search for new compounds of potential pharmaceutical interest, we focused our interest on *Papaver rhoeas* L. (Papaveraceae, commonly named “poppy” or “corn poppy”), a common plant which grows in fields in many different regions globally. For a long time, it has been appreciated for its pharmaceutical properties to treat coughs, diarrhea, and sleep disorders; however, when used as food, a large amount of baked *P. rhoeas* can induce intoxication in humans [3]. *P. rhoeas* aerial parts have been investigated and the alkaloid component identified [4,5] and its antimicrobial activity tested [4]. *P. rhoeas* has also undergone detailed investigation in the study of the incompatibility process, proving to be a model to investigate such aspects of sexual plant reproduction [6].

In the present paper we reported our findings on extracts from *P. rhoeas* ovules. First, we carried out optical and scanning electron microscopy investigations of ovules within the ovary and then, after ovule isolation and ethanol extraction, the extracts were separated by thin-layer chromatography techniques. Extracts and obtained spots were further evaluated for their cytotoxic activity by methylthiazolyldiphenyl-tetrazolium bromide test (MTT test) on HL60 human leukemia cells. Positive spots were investigated by gas chromatography and mass spectrometry (GC-MS) and their chemical composition elucidated.

## 2. Results

### 2.1. Optical Microscopy and Scanning Electron Microscopy Investigations of P. rhoeas Ovary and Ovules

**Figure 1:**
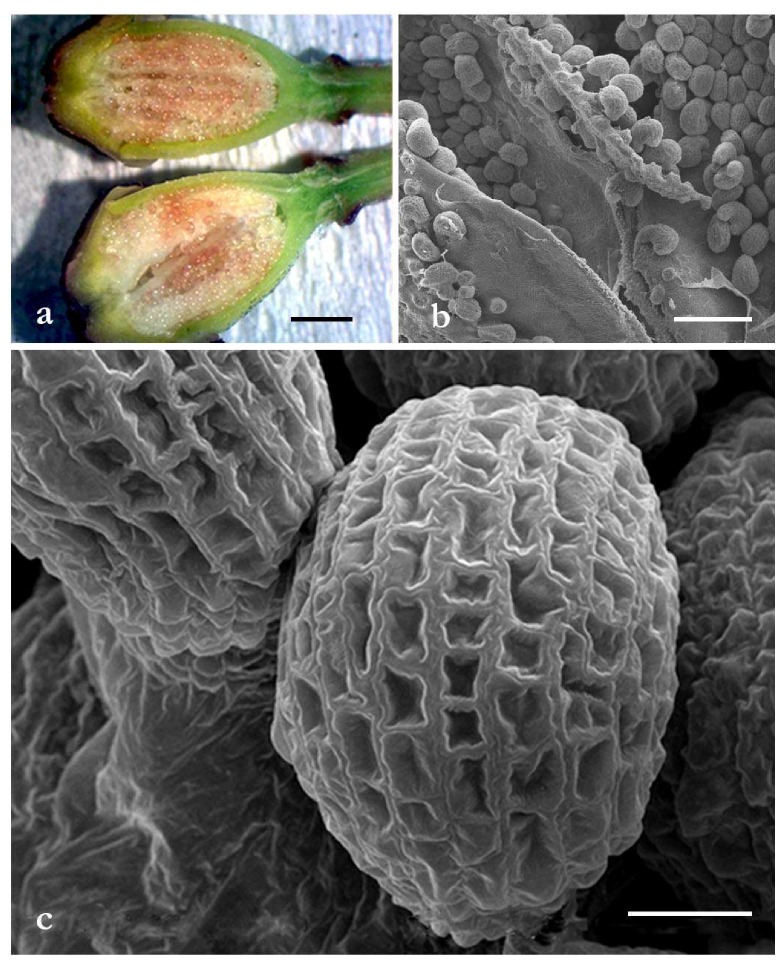
Ovules present inside the ovary observed by optical microscopy (**a**) and scanning electron microscopy (**b**). Ovules showed an elongated, round shape with a diameter of about 170 µm. Regular geometrical sculptures were present on the ovule surface (**c**). Bars: (**a**), 2 mm; (**b**), 500 µm; (**c**), 50 µm.

Images of ovary and ovules investigated by optical microscopy (OM) and scanning electron microscopy (SEM) are reported in Figure 1.

Many ovules were present inside the ovary with an ordered distribution (Figure 1a,b). The ovules showed an elongated, round shape with a diameter of about 170 µm. The ovule surface was covered by sculptures made up of regular geometrical spaces (Figure 1c).

### 2.2. Thin-Layer Chromatography Investigation and MTT Tests

When processed by TLC, the ovule ethanol extract formed eight well-separated UV-visible spots, which we progressively named S1 to S8 from the run starting line. The biological activity of the ovule ethanol extract and the TLC-obtained single spots were then tested on HL60 human leukemia cells (Table 1).

The biological activity of the ovule extract showed an Effective Concentration EC_50_ 119.233 ± 42.755 µg/mL, whereas the obtained fraction S1–S5 samples showed EC_50_ > 250 µg/mL and displayed no significant effects (data not shown). Interesting results were obtained for S6, S7, and S8 TLC-separated spots, showing EC_50_ 127.363 ± 41.932 µg/mL, 5.235 ± 1.501 µg/mL, and 12.100 ± 0.823 µg/mL, respectively (Table 1).

### 2.3. GC-MS Investigation of Spots Showing Cytotoxic Activity

Since there were interesting values of cytotoxic activity, S6, S7, and S8 samples were further processed by GC-MS techniques and their chemical composition investigated (Table 2). All data were expressed as mean ± SD. Except for three compounds (1-hexanol, 2-ethyl-; 2-mercaptoethanol; diethylene glycol hexyl ether) present in both S6 and S7 samples and one compound (hexadecanoic acid, ethyl ester) present in S7 and S8 samples, the fractions consisted of specific compounds, thus confirming a good level of sample separation obtained by TLC. S6 consisted of five different compounds with the 2-ethylhexyl fumarate as its main component (53.90%). Two other compounds, 2-mercaptoethanol and diethylene glycolhexyl ether, were present, with 15.00% and 21.95% of the total spot composition, respectively.

S7 consisted of 10 compounds, two of which, dipalmitin (22.30%) and 9,12-octadienoic acid, ethyl ester (22.50%), represented the main components followed by 9-octadecenamide, (*Z*)- (17.15%). In S8 nine compounds were identified and 2,6-di-tert-butylphenol (29.95%) and elaidic acid, methyl ester (22.15%) were the most abundant followed by hexadecanoic acid, ethyl ester (15.90%)

## 3. Discussion

In many places in the world, *P. rhoeas* flowers color the landscape with their characteristic red petals, and historically the large availability of such flowers met human needs, with *P. rhoeas* being used in phytotherapy and also as food [3].

Our microscopy investigations showed a large number of ovules within the ovary. The ovules had similar diameter and an elongated, roundish shape. Their surface presented sculptures, organized in a well-ordered and robust network, whose possible role could be related to the protection and preservation of the ovule’s content.

Our investigations then clearly reported that some ovule TLC-obtained fractions, named S6, S7, and S8, respectively, showed interesting cytotoxic properties when tested on HL60 human leukemia cells. An intense cytotoxic activity after administration of very low spot content concentrations was observed, thus opening further perspectives for chemical investigation.

GC-MS analysis of the S6, S7, and S8 fractions revealed the presence of 20 phytochemical compounds. Such a rich chemical composition did not allow analysis to relate the tested cytotoxic activities to single compounds. However, it could be possible that a synergistic cooperation of compounds consisting of isolated spots can occur, inducing the high mortality of HL60 cells observed.

*P. rhoeas* extract activity was tested on some cancer cell lines and no significative results were obtained [7,8]. *P. rhoeas* flower extract was studied on TK6 human lymphoblastoid cell line and antioxidant and cytotoxic effects were observed only at high concentrations [9].

Some of the identified compounds have also been found in other plant samples and their biological properties partially investigated. Dibutyl phthalate was present in fraction S6 (4.00%). This compound is a phthalate derivate and, applied by industry as a plasticizer, can have effects on human health. Uncontrolled waste disposal could release phthalate derivatives in water and soil, resulting in absorption by herbaceous plants or medicinal plants [10] and the extracts and essential oils of the plants that have been exposed to phthalates will be rich in these plasticizers. Dipalmitin, found in the S7 fraction, was also present in a chloroform fraction of *Acacia nilotica* L. leaves [11]. The 9-Octadecenamide, (*Z*)- (also known as oleamide) was found in the S7 fraction (17.15%). The fatty acid amide family includes anandamide and oleamide. Oleamide is being studied as a potential medical treatment for mood and sleep disorders and cannabinoid-regulated depression. Mikautadze et al. [12] reported that oleamide also possesses anti-epileptic features and significantly decreases the degree of convulsions induced by pentylentetrazole in rats.

The 2-Mercaptoethanol was present in fractions S6 and S7 (15.00% and 1.38%. respectively). Mattern et al. [13] reported its action as a reducing agent on aerobe and anaerobe broth cultures selectively. The 2,4-di-tert-butylphenol and 2,6-di-tert-butylphenol were found in fractions S7 (2.50%) and S8 (29.95%), respectively. As reported by different research papers [14,15], the first compound has a cytotoxic activity against human cancer cell lines, (KB, MCF7, A549, CasKi, and Hela). Furthermore, its antifungal activity against *Candida albicans* has been described [16]. The cytotoxic and radical scavenging activities of 2,6-di-tert-butylphenol were investigated by Kadoma et al. [17], highlighting the mechanism of action.

In Table 1, some compounds belonging to the fatty acids class and their esters are reported. Only fraction S8 showed the presence of elaidic acid, methyl ester (22.15%). This compound, also founded in Iranian olive fruit oil [18], is an esterified form of elaidic acid (fatty acid), which in turn is the trans isomer of oleic acid. Abbey and Nestel [19] reported that elaidic acid increased low density lipoprotein cholesterol (LDL) and decreased high density lipoprotein cholesterol (HDL) in humans.

Hexadecanoic acid, methyl ester was present in the S8 fraction (3.15%), while hexadecanoic acid, ethyl ester was found in the S7 (2.65%) and S8 (15.90%) fractions. The fatty acid methyl ester had antifungal, antioxidant, and antibacterial properties [20]; for the ethyl ester, an antioxidant property was reported [21].

In the S8 fraction, n-hexadecanoic acid (palmitic acid) was found (11.75%) and the bioactivity reported regarded anti-inflammatory [22] and mosquito larvicide [23] properties. Moreover, Ravi et al. [24] extracted n-hexadecanoic acid from *Kigelia pinnata* leaves and proposed that the cytotoxic activity was due to its interaction with DNA topoisomersi I.

The 9,12-Octadienoic acid, ethyl ester (linoleic acid ethyl ester) found in the fraction S7 (22.5%) has been shown to be a compound with many biological activities, such as hypocholesterolemic, hepatoprotective anti-androgenic, antihistaminic, antiezemic, and antiacne activities, as well as being a 5-alpha reductase inhibitor [25].

## 4. Materials and Methods

### 4.1. Sample Preparation and Optical Microscopy and Scanning Electron Microscopy Investigations of P. rhoeas Ovules

*P. rhoeas* flowers were collected at the Botanical Garden “Angelo Rambelli” of Tuscia University, Viterbo, and the flowers processed in our laboratory under observation with a stereo microscope. Petals and stamens were removed and the pistils excised using a razor blade. The ovaries and their ovule content were observed using a stereo microscope and some images were taken. Samples were then processed for SEM investigation.

For SEM observations, ovaries containing ovules were collected in tubes, washed with phosphate buffered saline, and fixed with 4% paraformaldehyde and 5% glutaraldehyde, pH 7.2 in 0.1 M cacodylate buffer for 1 h at 4 °C [26]. After rinsing overnight in the same buffer, samples were post-fixed in 1% osmium tetroxide in cacodylate buffer for 1 h at 4 °C. After two washings in the same buffer, samples were dehydrated in a graded ethanol series. Ovaries containing ovules were dried using the critical point drying method with CO_2_ in a Balzers Union Critical Point Dryer (CPD) 020, sputter-coated with gold in a Balzers MED 010 unit and observed with a JEOL JSM 5200 Scanning Electron Microscope.

### 4.2. P. rhoeas Ovule Extract Preparation and Thin-Layer Chromatography (TLC) Investigations

For extraction of compounds, ovules were collected from the ovaries using a rubber policeman, and added to a tube containing 1 mL of ethanol. Ovules were then homogenated for 10 min on ice by a Potter apparatus and the solution obtained was centrifuged at 14,000 rpm for 5 min by a microfuge. The pellet was discarded and the supernatant dried. The residual powders were weighed and dissolved in ethanol. One sample aliquot was stored at 4 °C for further tests on HL60 human cells. Another sample aliquot was investigated by TLC on a 20X20 silica gel plate (Macherey-Nagel, Düren, Germany) and a mobile phase consisting of ethanol:chloroform (1:1). At the end of the run, the plate was air dried and observed under UV lamps. The separated spots were scraped, the contents solubilized by ethanol, and the obtained samples stored at 4 °C before being tested on HL60 human leukemia cells for evaluation of possible cytotoxic activities.

For the preparation of samples for biological tests, the ethanol *P. rhoeas* ovule extract and TLC-separated spots were concentrated 3 times in an evaporator, the residues resuspended in Milli-Q water (3 volumes), and concentrated again 3 times in order to avoid ethanol contamination. The resulting residues were resuspended in Milli-Q water (3 volumes), the obtained samples centrifuged at 14,000 rpm for 3 min, and the supernatants filtered through a 0.22-µm cellulose syringe filter and stored at −20 °C until testing.

### 4.3. HL60 Cell Culture and MTT Assay

HL60 human leukemia cells were cultured in Dulbecco’s Modified Eagle’s Medium (DMEM) supplemented with 10% heat-inactivated fetal bovine serum (FBS), 2 mM glutamine, 100 U/mL penicillin, and 100 µg/mL streptomycin at 37 °C in 5% CO_2_. To investigate the cytotoxic potential of the extract and the TLC-separated spots, the MTT colorimetric assay [27] was performed with some minor modifications. Briefly, 2 × 10^4^ cells suspended in 100 μL of growth medium were seeded in 96-well plates, and after 24 h, 100 μL of medium containing each sample previously dissolved in DMSO (final concentration 1% *v*/*v*) was added. The extract and the fractions were tested at serial dilutions (1:2) ranging from 500 to 0.97 µg/mL and from 62.5 to 0.12 µg/mL, respectively. Untreated and DMSO (1%)-treated cells were used as controls, while VBL (vinblastine sulfate, Sigma, Milano, Italy) was added as positive control from 10 µg/mL to 0.01 µg/mL). After 24 h, the media with and without drugs (control) was removed and 100 µL of MTT solution was added to each well and further incubated for 3 h at 37 °C in a 5% CO_2_ humidified atmosphere. Then 100 µL of DMSO was added to solubilize the resulting purple formazan crystals produced from metabolically viable cells. Absorbance (Abs) was measured by a Tecan microplate reader at 595 nm and seven wells were used for each sample assayed. Three independent experiments were performed. The percentage of cytotoxic activity was determined by the following formula: Cell viability (%) = [Abs of treated cells/Abs of untreated cells] × 100. Half-maximal effective concentration (EC_50_), required to have an effect on 50% of cell proliferation, was calculated from the mean values of data from each well by using the AAT Bioquest (Sunnyvale, CA, USA) EC_50_ calculator (https://www.aatbio.com/tools/ec50-calculator/). Statistical analyses were performed and the results expressed as mean ± SD. Data were analyzed with one-way analysis of variance (ANOVA) and a one-tailed unpaired test using GraphPad Prism software (GraphPad Prism 5.0, GraphPad Software, Inc., San Diego, CA, USA), with values ≤0.05 as statistically significant.

### 4.4. GC-MS Chemical Investigations

The TLC-separated spots showing significant cytotoxic activity were analyzed using a GC-MS Perkin Elmer Clarus 500 instrument equipped with a Restek Stabilwax fused-silica capillary column (length 60 m × 0.25 mm Internal Diameter (ID)). Helium was used as the carrier gas with a flow rate of 1 mL/min, and the oven temperature program was as follows: 5 min at 70 °C then a gradient of 6 °C/min to 250 °C for 30 min. The dried extracts were dissolved in 1 mL of CH_3_OH and 2 µL of the solution manually injected at 280 °C into the GC injector in the splitless mode. The mass spectrometer was scanned from mass/charge (*m*/*z*) 40–450 atomic mass unit (amu) with scan time 0.2 s. All mass spectra were recorded in the electron impact ionization (EI) at 70 eV. Relative percentages for quantification of the components were calculated by electronic integration of the Flame Ionization Detector (GC-FID) peak areas. The identification of the components was performed by comparison of their linear retention indices (LRIs) and spectral mass with those reported in library data (Wiley 02 and Nist) of the GC-MS system. The LRI of each compound was calculated using a mixture of aliphatic hydrocarbons (C_8_–C_30_, Ultrasci) injected directly into the GC injector at the same temperature program as detailed above. Relative abundances of the separated components were derived by using the same instrumentation with the FID detector configuration, without the use of either an internal standard or correction factors. Analyses were repeated under the same operative conditions for calculation of standard deviation of peak area percentage of each identified component in the chromatogram.

## 5. Conclusions

*P. rhoeas* ovules were investigated at morphological, chemical, and biological level and new findings were reported. The presence of compounds showing cytotoxic activity on HL60 human cells opens interesting perspectives for further investigation on other human cancer cell lines.

## Figures and Tables

**Table 1:** Biological activity was expressed in Effective Concentration EC_50_ values ± SD.

Samples	EC_50_ µg/mL (Mean ± SD)
Extract	119.233 ± 42.755
S6	127.363 ± 41.932
S7	5.235 ± 1.501
S8	12.100 ± 0.823
VBL	0.019 ± 0.002

**Table 2:** Chemical composition (%) of S6, S7, and S8 samples obtained by thin-layer chromatography separation from *Papaver rhoeas* ovule ethanol extract.

Component	LRI ^1^	LRI^lit 2^	S6 (%)	S7 (%)	S8 (%)
hexanal	1075	1081	-	-	0.80 ± 0.14
2-heptenal, (*Z*)-	1280	1287	-	-	1.78 ± 0.03
3,5-octadien-2-ol	1480	*	-	-	10.40 ± 0.14
1-hexanol,2-ethyl-	1491	1494	3.93 ± 0.03	0.68 ± 0.03	-
2-mercaptoethanol	1495	1498 °	15.00 ± 0.28	1.38 ± 0.03	-
hexacosane	1505	*	-	7.90 ± 0.14	-
2-decenal, (*E*)-	1645	1650	-	-	3.40 ± 0.28
hexadecanoic acid, methyl ester	2246	2251	-	-	3.15 ± 0.21
hexadecanoic acid, ethyl ester	2280	2288	-	2.65 ± 0.21	15.90 ± 0.14
2,4-di-tert-butylphenol	2317	2321	-	2.50 ± 0.14	-
2,6-di-tert-butylphenol		2327 ^+^	-	-	29.95 ± 0.21
diethylene glycol hexyl ether	2400	*	21.95 ± 0.21	12.40 ± 0.14	-
elaidic acid, methyl ester		2445 ^+^	-	-	22.15 ± 0.35
9,12-octadienoic acid, ethyl ester	2555	2560	-	22.50 ± 1.41	-
dibutyl phthalate	2610	2618	4.00 ± 0.14	-	-
*n*-hexadecanoic acid	2920	2928	-	-	11.75 ± 0.21
dodecanoic acid, 3-hydroxy	3000	*	-	8.90 ± 0.28	-
9-octadecenamide, (*Z*)-	3260	3265 ^+^	-	17.15 ± 0.49	-
2-ethylhexyl fumarate	3300	*	53.90 ± 1.13	-	-
dipalmitin	3500	*	-	22.30 ± 0.56	-
**Sum**			98.78	98.36	99.28

^1^ Linear Retention Indices (LRI) measured on polar column; ^2^ LRI from literature; * LRI^lit^ not available; ^+^ Normal alkane RI; ° LRI for custom T program.
